# Deep Learning-Based System for Preoperative Safety Management in Cataract Surgery

**DOI:** 10.3390/jcm11185397

**Published:** 2022-09-14

**Authors:** Gaku Kiuchi, Mao Tanabe, Katsunori Nagata, Naofumi Ishitobi, Hitoshi Tabuchi, Tetsuro Oshika

**Affiliations:** 1 Department of Ophthalmology, Faculty of Medicine, University of Tsukuba, 1-1-1 Tennoudai, Tsukuba 305-8575, Japan; 2 Department of Ophthalmology, Tsukazaki Hospital, 68-1 Waku, Aboshi-ku, Himeji 671-1227, Japan; 3 Department of Technology and Design Thinking for Medicine, Hiroshima University, 1-2-3 Kasumi, Minami-ku, Hiroshima 734-8551, Japan

**Keywords:** artificial intelligence, deep learning, cataract surgery, preoperative safety management, facial recognition, laterality, intraocular lens

## Abstract

An artificial intelligence-based system was implemented for preoperative safety management in cataract surgery, including facial recognition, laterality (right and left eye) confirmation, and intraocular lens (IOL) parameter verification. A deep-learning model was constructed with a face identification development kit for facial recognition, the You Only Look Once Version 3 (YOLOv3) algorithm for laterality confirmation, and the Visual Geometry Group-16 (VGG-16) for IOL parameter verification. In 171 patients who were undergoing phacoemulsification and IOL implantation, a mobile device (iPad mini, Apple Inc.) camera was used to capture patients’ faces, location of surgical drape aperture, and IOL parameter descriptions on the packages, which were then checked with the information stored in the referral database. The authentication rates on the first attempt and after repeated attempts were 92.0% and 96.3% for facial recognition, 82.5% and 98.2% for laterality confirmation, and 67.4% and 88.9% for IOL parameter verification, respectively. After authentication, both the false rejection rate and the false acceptance rate were 0% for all three parameters. An artificial intelligence-based system for preoperative safety management was implemented in real cataract surgery with a passable authentication rate and very high accuracy.

## 1. Introduction

The term wrong-site surgery usually encompasses operations on the wrong patients, performing the wrong surgery (other than the one indicated or intended), or performing surgery on the wrong body part or, for body parts that are symmetric, on the wrong side [1]. Even with the introduction of mandatory surgical checklists [2,3,4] and widespread implementation of the Universal Protocol [5], wrong-site surgery continues to pose significant challenges to patient safety [6].

In ophthalmic procedures, common surgical confusions include incorrect intraocular lens (IOL) implantation, incorrect eye procedures (laterality error), and incorrect patients or procedures [7,8,9]. Wrong-site errors result from misinformation or misperception [10]. Misperception can stem from laterality or location confusion, particularly with confirmation bias, which is the psychological tendency to confirm a mental impression instead of the physical facts [10]. The key to preventing wrong-site surgery is to have multiple independent checks of critical information; thus the addition of artificial intelligence (AI), which is free from confirmation bias and hasty judgment, to the check system may be beneficial to prevent “never-events” in the operating room. We conducted the current study to investigate the efficacy and feasibility of AI-based systems to reduce the risk of wrong-site operations in cataract surgery.

## 2. Materials and Methods

### 2.1. Artificial Intelligence System

An iOS-based app was developed by the Tsukazaki Hospital AI team for facial recognition of patients, laterality confirmation (right or left eye), and IOL parameter verification.

The facial recognition system involving two main tasks, face detection and recognition, was constructed based on the facial recognition development kit (ISG-539, Glory Ltd., Hyogo, Japan). Prior to the day of surgery, a face photo of the patient not wearing a mask was taken with a mobile device (iPad or iPad mini, Apple Inc., Cupertino, CA, USA) camera and stored in the referral database. The mobile device was used just as a camera without using the facial recognition function of iOS. Approximately 100 facial key points were utilized for identification of individual faces. On the day of surgery, before entering the operating room, the patient’s face photo without a mask was captured with a mobile device camera, which was then checked with the reference photograph stored in the database.

**Figure 1:**
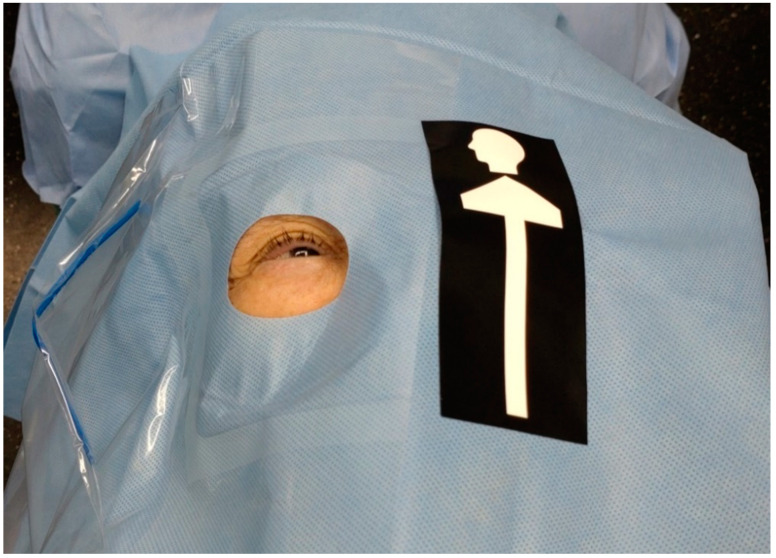
Surgical drape placed over the patient. The right eye is exposed through the aperture and a black arrow sticker indicating the head direction is pasted over the contralateral eye on the drape.

The laterality confirmation system was developed with a two-stage object detection model of the You Only Look Once Version 3 (YOLOv3) algorithm [11,12]. This system was trained to recognize the relative location of the aperture of a surgical drape and direction of an arrow pasted over the contralateral eye on the surgical drape (Figure 1). In the first stage, the model identifies the arrow on the sticker indicating the head direction. In the second stage, the model recognizes the right/left side of the surgical drape aperture. A total of 1171 images were used to train the YOLOv3 model for the first stage (momentum = 0.9, decay = 0.000001, learning rate = 0.001), and 1167 images were used to train the model for the second stage (momentum = 0.9, decay = 0.000001, learning rate = 0.001). In the operating room, after placing the surgical drape and arrow sticker over the patient on the operating table, a photo was taken with the mobile device camera and the AI application was run to verify the laterality. The photo can be taken from any direction: head, foot, right, or left side of the patient.

**Figure 2:**
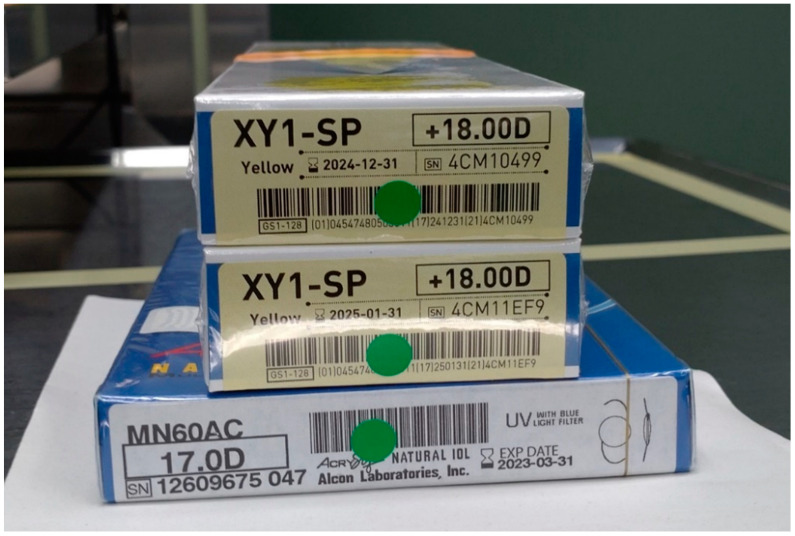
Intraocular lens packages. A mobile device camera is used to capture the image to recognize the parameters shown on the packages of planned, reserved, and backup lenses.

The IOL parameter verification system was constructed and trained in three stages: detection of the information area on IOL packages by YOLOv3, identification of the IOL model number by the Visual Geometry Group-16 (VGG-16) architecture [13,14], and recognition of IOL power by another VGG-16. The YOLOv3 model was trained with 1732 images (momentum = 0.9, decay = 0.0005, learning rate = 0.001). The VGG-16 model for identifying the IOL model number was trained with 123,616 IOL images (momentum = 0.001, decay = 0.000001, learning rate = 0.001). The VGG-16 model for recognition of IOL power was trained with 57,126 images (momentum = 0.001, decay = 0.000001, learning rate = 0.001). Twenty two types of IOLs from 3 manufacturers were used for the training. In the operating room, the mobile device camera was used to capture the image of IOL packages including the planned, reserved, and backup IOLs (Figure 2). The IOL parameters were recognized by the AI app, which were then checked with the information registered in the database.

### 2.2. Patients

A total of 171 patients who were undergoing phacoemulsification and IOL implantation at the Tsukuba University Hospital between February and May 2022 were included. There were 67 males (91 eyes) and 55 females (80 eyes), and their age averaged 66.8 ± 16.0 (range, 4~97) years. Due to partial consent given by some patients and incomplete AI training for new IOLs, different numbers of patients participated in the three trials: 162 patients in the facial recognition test, 171 in the laterality check, and 135 in the IOL parameter verification.

The authentication rate on the first attempt, the cumulative authentication rate after repeated attempts, false rejection rate, and false acceptance rate were evaluated. Eighteen clinicians took part in the photographing and authentication processes. One patient was verified by a single clinician.

The Institutional Review Board of the Tsukuba University Hospital approved the study protocol (R02-252). This study was conducted in accordance with the Declaration of Helsinki. Informed consent for data acquisition was obtained from all subjects involved in the study.

## 3. Results

**Figure 3:**
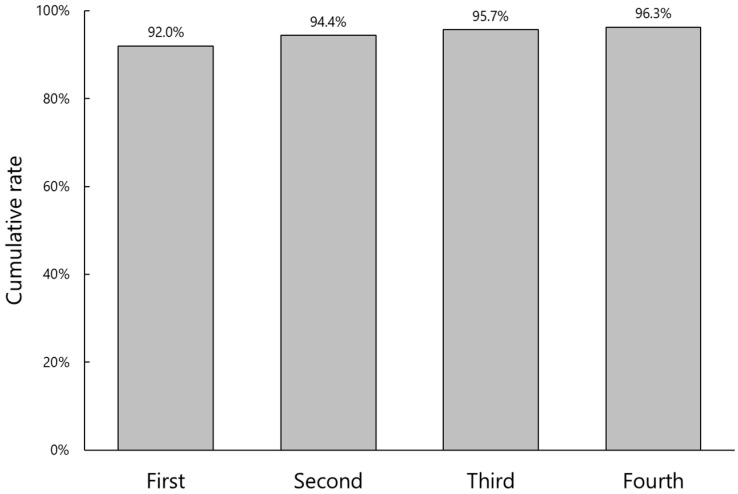
Cumulative authentication rate of facial recognition against number of attempts.

The cumulative authentication rate and number of attempts for facial recognition are shown in Figure 3. Facial recognition was successful in 92.0% on the first attempt, and the rate increased to 96.3% after the fourth attempt. In six eyes (3.7%), the AI system failed to identify the patient’s face even after repeated attempts. Among them, pre-captured face photos in the referral database were not adequate in four cases, i.e., the patients wore a mask or photos were taken from the side rather than being frontal. In two cases, the patient’s face was out-of-frame in the photo taken before entering the operating room.

**Figure 4:**
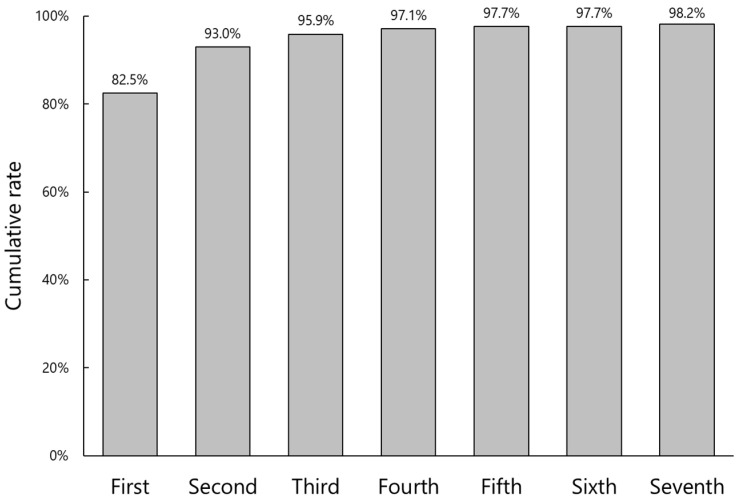
Cumulative authentication rate of laterality (right or left eye) confirmation against number of attempts.

Laterality (right or left eye) confirmation was successful in 82.5% on the first attempt, which increased to 98.2% after the seventh trial (Figure 4). In three cases (1.8%), the laterality authentication was not achieved due to poor shooting conditions.

**Figure 5:**
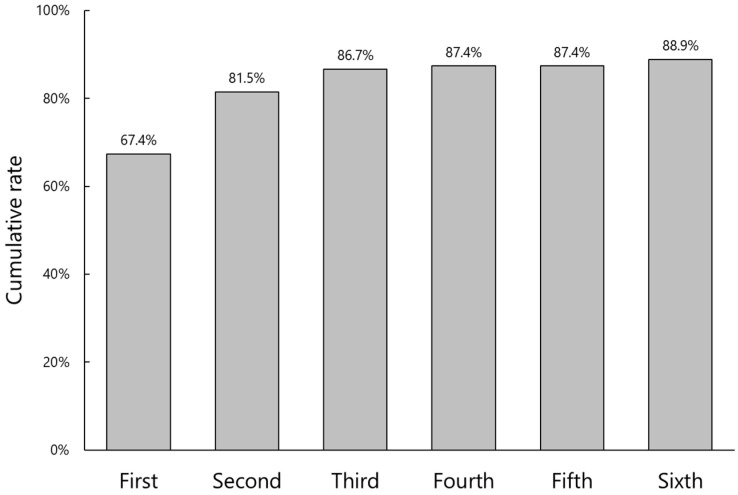
Cumulative authentication rate of intraocular lens parameter verification against number of attempts.

**Figure 6:**
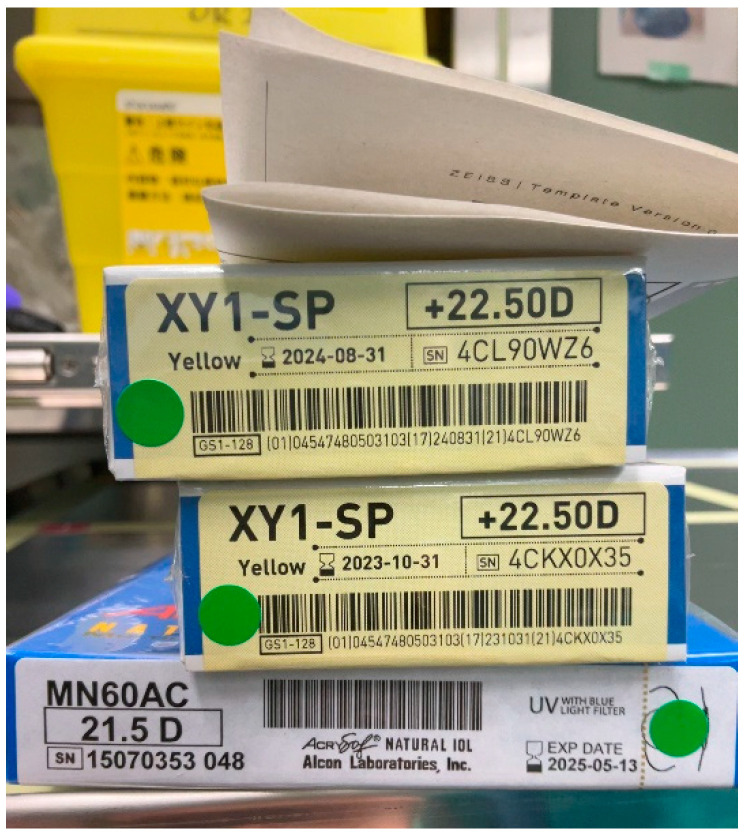
An example of poor shooting conditions. Unnecessary documents placed on the package and character information in the background hamper accurate recognition of intraocular lens parameters.

IOL parameter verification was more difficult with low authentication rates even after several attempts (Figure 5). The initial and final authentication rates were 67.4% and 88.9%, respectively. In 15 cases (11.1%), IOL parameters could not be verified. The reasons for failure included an incomplete IOL referral database, especially that of the latest model of IOL, inaccurate registration of backup IOLs to the patient file, and poor shooting conditions (Figure 6).

After authentication, both the false rejection rate and the false acceptance rate were 0% for all three parameters: facial recognition, laterality confirmation, and IOL parameter verification.

## 4. Discussion

In the current study, we evaluated the authentication rate and accuracy of the AI-based app for facial recognition, laterality confirmation, and IOL parameter verification in cataract surgery. Once the app recognized a patient’s face, eye, or IOL parameters, both the false rejection rate and the false acceptance rate were 0% for all three parameters, indicating the high accuracy of this identification system. On the other hand, the authentication rate was not satisfactory, especially for IOL parameter verification.

Facial recognition technology has been increasingly applied in the healthcare industry for its perceived accuracy in identifying individuals and has been proposed as one of the reliable ways to improve patient security. Especially with the COVID-19 pandemic in recent years, facial recognition technology has a significant advantage compared to other methods of identification; it doesn’t require any physical contact and can be used from a distance. Besides simplifying the check-in and check-out process and improving the overall patient experience, facial recognition helps prevent fraud and misidentification of patients. In the current study, facial recognition failed in six cases (3.7%). The reasons for failure include inadequate face photos registered in the referral database and poor photographing before surgery, both of which are human errors and can be avoided with due diligence.

We employed the YOLOv3 algorithm for laterality confirmation. A similar deep learning method has been used to distinguish the right and left foot using plantar pressure images [15]. Since the right and left footprints have mirror images, their classification is rather easy. On the other hand, it is difficult for an app to recognize right and left eyes with the patient’s face covered by a surgical drape. We conducted a pilot study in which the AI-based app judged the laterality of the eye based only on the relative location of the aperture of a surgical drape. The authentication rate on the first attempt was quite low with only 75.4% (unpublished data). In addition, the photos had to be taken from the head-side of the patients, making the traffic line in the operation room quite complicated. We then introduced an arrow sticker indicating the head direction of patients for better recognition (Figure 1). As a result, the photos could be taken from any directions, and the authentication rate on the first attempt and after repeated attempts improved to 82.5% and 98.2%, respectively. In order to further raise the initial authentication rate, we are trying to implement more efficient and reproducible ways to capture the target images.

Incorrect IOL implantation is the leading cause of medical mishaps in cataract surgery [7,8,9]. Actually, the current study indicated that IOL parameter verification was the most challenging task for our AI-based app system, with the lowest initial and final authentication rates. The reasons for failure include an incomplete IOL referral database, especially that of the latest model of IOL, inaccurate registration of backup IOLs to the patient file, poor shooting conditions, and others. We have been working to resolve these problems, and actually the authentication rate for IOL parameter verification has improved during the four months of the current study from 78.4 in February to 91.2% in May 2022 (unpublished data).

It is obvious that the AI-based system cannot completely replace the current human checklist system for surgical information confirmation. As shown in this study, the authentication rate of the app is not and will not be 100%, and the referral database cannot always be perfect. In particular, the IOL verification system needs to be trained and updated regularly when a new IOL is introduced or even when the package design is modified. In addition, the patient profile database including face photo and information, which is created and maintained by humans, may be defective. Nevertheless, the human checklist system is not flawless, either, and thus the AI-based app system is considered to be a powerful safety-net guarantee to help reduce thoughtless mistakes. The performance of an AI-based confirmation system, involving photo shooting with an iPad or iPad mini, adds a few seconds to the “time-out” duration in the operating room, and the implementation of a new system requires additional costs. Once implemented, however, the AI-system is tireless and consistent, and its running cost is far lower than the salary for a human medical staff. Once surgical confusion occurs, its consequences can be devastating. Introduction of a reliable AI-based system would be beneficial in terms of related time- and cost-effectiveness.

There are several limitations to this study. First, the current study has no control group. It is ideal to compare the data before and after the implementation of our AI-based app system to prove its efficacy. With an extremely low incidence of surgical confusion in the real world, however, it is difficult to quantitatively demonstrate the differences before and after implementation. Second, the subjects of our study were not consecutive cases. Some patients refused to participate, and others consented to participate in a part of the study; specifically, several patients declined to have their faces photographed. Participants and non-participants were randomly mixed in our study population. This was one of the reasons why the study was confused especially for IOL parameter verification. Third, we only assessed the authentication rate and accuracy of three parameters, and other factors were not evaluated, such as time and effort required for the implementation of this system. We suppose that additional time and effort are needed in the early period of system introduction, but the workflow will eventually be improved when staff members become more familiar with the process, making the preoperative safety management more precise and smoother than the conventional analog approaches.

## 5. Conclusions

We implemented a deep learning-based system for preoperative safety management in cataract surgery. The final authentication rate for facial recognition, laterality (right and left eye) confirmation, and IOL parameter verification was 96.3%, 98.2%, and 88.9%, respectively. After authentication, both the false rejection rate and the false acceptance rate were 0% for all three parameters.

## Data Availability

The datasets generated during and/or analyzed during the current study are available from the corresponding author on reasonable request.
